# Polymorphisms in the H19 gene and the risk of lung Cancer among female never smokers in Shenyang, China

**DOI:** 10.1186/s12885-018-4795-6

**Published:** 2018-09-15

**Authors:** Zhihua Yin, Zhigang Cui, Hang Li, Juan Li, Baosen Zhou

**Affiliations:** 1 Department of Epidemiology, China Medical University, No. 77, Puhe Road, Shenyang North District, Shenyang, 110122 People’s Republic of China; 2 School of Nursing, China Medical University, Shenyang, 110122 People’s Republic of China

**Keywords:** Lung cancer, H19, Single nucleotide polymorphism, Cooking oil fume, Interaction

## Abstract

**Background:**

Long non-coding RNA (lncRNA) H19 is a hot spot in tumor development, progression and metastasis. This study assessed the association between H19 genetic polymorphisms and the susceptibility of lung cancer.

**Methods:**

The case-control study was conducted to evaluate the association between four selected single nucleotide polymorphisms (rs217727, rs2107425, rs2735469 and rs17658052) in H19 gene and the risk of lung cancer. There were 556 female never smoking lung cancer patients and 395 cancer-free controls. Unconditional logistic regression analysis was used to analyze the associations between four SNPs and lung cancer risks by calculating the odds ratios and their 95% confidence intervals. The gene-environment interactions were assessed on both additive and multiplicative scales.

**Results:**

Compared with carriers carrying homozygous CC genotype, there was a statistically significant increased risk of lung cancer for carriers of the rs2107425 TT genotype (odds ratio = 1.599, 95%CI = 1.106–2.313, *P* = 0.013). In both dominant and recessive models, significant associations were found between rs2107425 and lung cancer risk, and the corresponding odds ratios were 1.346 (1.022–1.774) and 1.400 (1.011–1.937), with *P* values 0.035 and 0.043, respectively. There was no significant correlation between lung cancer risk and rs2735469, rs217727 and rs17658052. Interaction analysis showed that their combined effects had a greater impact on lung cancer than individual effects of polymorphism and cooking smoke exposure. However, further analysis showed that the both additive model and the multiplicative model were not statistically significant.

**Conclusion:**

The polymorphism rs2107425 in H19 gene was associated with the risk of lung cancer among female who never smokes in Shenyang, China.

**Electronic supplementary material:**

The online version of this article (10.1186/s12885-018-4795-6) contains supplementary material, which is available to authorized users.

## Background

Since 1985, lung cancer has been the leading cause of cancer-related deaths worldwide [1, 2]. According to reports, the incidence and mortality of lung cancer are the highest among various types of cancer in both urban and rural areas of China [3]. Smoking is the most important risk factor for lung cancer. However, global statistics suggest that the cause of lung cancer in 15% of men and 53% of women cannot be attributed to smoking [4]. Therefore, other risk factors might also be important in the development of lung cancer. Despite the wide geographical differences, never-smoking lung cancer patients are more common in women [5]. Therefore, it is urgent to explore the risk factors of lung cancer in female never smokers.

Molecular epidemiological studies play a key role in studying the genes involved in lung cancer. In recent years, molecular mechanisms associated with lung cancer may be revealed by newly developed markers such as non-coding RNAs (ncRNAs). Long noncoding RNAs (lncRNAs), the largest family of non-coding transcripts, play key roles in regulating chromatin dynamics, gene expression, growth, differentiation, and development [6]. lncRNA has been found to be abnormally expressed or mutated in cancer in transcriptome studies using next-generation sequencing in recent years [7].

H19, located in a cluster with the insulin-like growth factor 2 (IGF2) gene on chromosome 11p15.5, is one of the most important lncRNAs. H19 acts as a gene that is up-regulated in hypoxic stress and certain tumors, including lung cancer, and is therefore an indispensable regulator of tumor development [7–12]. Kaplan et al. observe that the expression of H19 in airway epithelial cells in non-smokers is lower than that in smokers [13]. Thereby, the up-regulation of airway epithelial H19 expression can be considered as an early marker of epithelial cell development into lung cancer. Barsyte-Lovejoy et al. have found that the Myc oncogene lead to H19 upregulation by specifically binding to the H19 promoter region, and also observed the strong relationship between H19 and c-MYC expression levels in lung cancer cells [14].

In recent years, single nucleotide polymorphisms (SNPs) of candidate genes have become the focus of many studies on the genetic susceptibility of cancer. The expression or function of the host lncRNA changes may due to sequence variation of the non-coding RNA gene. Now the researchers conclude that SNPs or mutations in lncRNA sequences may alter expression, and/or influence miRNA binding, and consequently lead to modified cancer risk [6–9]. However, the studies on the association between the SNPs in lncRNAs and the susceptibility of lung cancer are few so far. In this study, we genotyped four tag SNPs of H19 gene (rs217727, rs2107425, rs2735469, and rs17658052) in a case-control study of lung cancer in northeast China. To the best of our knowledge, this is the first study of lung cancer in a never-smoking female population, to assess the impact of SNPs in lncRNA H19 on lung cancer risk.

## Methods

This hospital-based case-control study was carried on in Shenyang City, which is in the northeast of China. The case group includes 556 newly histologically diagnosed lung cancer patients, who are all never smoking females. A total of 395 cancer-free controls is recruited from the medical examination centers and they are also female never smokers and matched to cases by age (±5 years). Individuals who smoke more than 100 cigarettes in their whole life are defined as smokers, otherwise they are considered as never smokers. Each subject donates 5 ml blood to detect the SNPs and completes a questionnaire including the basic characteristics and the exposure status of environmental factors. The admission of subjects, the epidemiological investigation of environmental factors and detecting of SNPs were performed according to our previous report [15]. Each participant has signed the informed consent form. The study was approved by the Institutional Review Board of the China Medical University.

Genomic DNA samples were obtained using Phenol-chloroform Method. Genotyping method of the studied SNPs was introduced in our previous paper [16]. In our previous studies, the significant association between the risk of lung cancer and cooking fume exposure in female never-smokers have been found. Thus, in this study, besides the study of H19 SNPs, we analyzed the interaction between these SNPs and cooking fume exposure on the risk of lung cancer.

The difference of age between cases and controls was assessed by t-test. Hardy-Weinberg equilibrium (HWE) of the genotypes was measured by a goodness-of-fit χ2 test. The associations between the SNPs and lung cancer risks were analyzed through unconditional logistic regression by calculating the odds ratios (OR) and their 95% confidence intervals (CI). Both additive model and multiplicative model were used to assess the interaction between the SNPs and cooking fume exposure. The additive interaction was stated through calculating three indicators, which were the relative excess risk due to interaction (RERI), the attributable proportion due to interaction (AP), and the synergy index (S). All of the statistical analyses were two-sided. The statistically significant level was defined as 0.05. The statistical analyses were performed using SPSS software (Version 20.0; IBM SPSS, Inc., Chicago, IL, USA).

## Results

The study was composed of 556 cases and 395 controls, who were all female never smokers in Shenyang, China. The mean ages for cases and controls were 56.74 ± 11.70 and 56.13 ± 11.64 years, with no statistically significant difference (*t* = − 0.797, *P* = 0.426). Of the 556 lung cancer cases, 371 (66.7%) patients were adenocarcinoma, 96 (17.3%) were squamous cell lung cancer and 89 (16.0%) were other types. Among the cases and controls, 100 and 66 people had a history of exposure to cooking fumes, respectively, with a statistically significant difference (χ2 = 9.739, *P* = 0.002). Individuals exposed to cooking fumes have an increased risk for female never smokers, and the corresponding odds ratio (OR) was 1.804, 95% confidence interval (CI) was 1.243–2.618. The observed genotype frequencies of four polymorphisms (rs217727, rs2107425, rs2735469 and rs17658052) were in agreement with that expected under the Hardy-Weinberg equilibrium in the controls (*P* values were 0.232, 0.513, 0.343 and 0.533, respectively).

Table 1 shows the relationship between four polymorphisms and lung cancer risks. Compared with CC genotype, rs2107425 TT genotype carriers had a statistically significant increase in lung cancer risk (adjusted OR was 1.599, 95%CI = 1.106–2.313, *P* = 0.013). There were also statistically significant results in both dominant model (CT + TT vs CC) and recessive model (CC vs CT + TT), and the corresponding ORs (95%CI) were 1.346 (1.022–1.774) and 1.400 (1.011–1.937), respectively. The T allele of rs2107425 was suggested to be risk allele of lung cancer (OR = 1.275, 95%CI = 1.060–1.533, *P* = 0.010). The similar significant associations were also found between rs2107425 with lung adenocarcinoma and squamous cell lung cancer (Table 2). In addition, the rs17658052 AG genotype was found to be significantly associated with an increased risk of squamous cell lung cancer (adjusted OR was 2.411, 95%CI = 1.175–4.946, *P* = 0.016). The A allele of rs17658052 was appeared to be related with the higher risk of squamous cell lung cancer compared with G allele (OR = 2.705, 95%CI = 1.390–5.262, *P* = 0.002). However, no significant associations were found for rs217727 and rs2735469 polymorphisms with lung cancer risks (Table 1 and Table 2).

**Table 1:** Association between H19 SNPs and lung cancer risks

H19		Controls (%)	Cases (%)	OR (95% CI)	*P* value	OR (95% CI)*	*P* value
rs217727	CC	165(41.8)	204(36.7)	1.00(ref)		1.00(ref)	
	CT	172(43.5)	264(47.5)	1.241(0.937–1.644)	0.132	1.247(0.941–1.652)	0.124
	TT	58(14.7)	88(15.8)	1.227(0.831–1.812)	0.303	1.229(0.832–1.816)	0.300
	CT + TT vs CC			1.238(0.951–1.612)	0.113	1.243(0.954–1.618)	0.107
	TT vs CT + CC			1.093(0.762–1.566)	0.630	1.092(0.762–1.565)	0.632
	C allele	502(63.5)	672(60.4)	1.00(ref)			
	T allele	288(36.5)	440(39.6)	1.141(0.945–1.378)	0.169		
rs2107425	CC	140(35.4)	161(29.0)	1.00(ref)		1.00(ref)	
	CT	185(46.8)	266(47.8)	1.250(0.932–1.678)	0.137	1.251(0.932–1.678)	0.136
	TT	70(17.7)	129(23.2)	1.602(1.108–2.317)	0.012	1.599(1.106–2.313)	0.013
	CT + TT vs CC			1.347(1.022–1.775)	0.034	1.346(1.022–1.774)	0.035
	CC vs CT + TT			1.403(1.014–1.941)	0.041	1.400(1.011–1.937)	0.043
	C allele	465(58.8)	588(52.9)	1.00(ref)			
	T allele	325(41.1)	524(47.1)	1.275(1.060–1.533)	0.010		
rs2735469	CC	359(90.9)	507(91.2)	1.00(ref)		1.00(ref)	
	CT	36(9.1)	46(8.3)	0.905(0.573–1.428)	0.668	0.902(0.571–1.425)	0.659
	TT	0(0)	3(0.5)	#	#	#	#
	CT + TT vs CC			0.964(0.614–1.513)	0.873	0.962(0.613–1.510)	0.866
	TT vs CC + CT			#	#	#	#
	C allele	754(95.4)	1060(95.3)	1.00(ref)			
	T allele	36(4.6)	52(4.7)	1.027(0.665–1.588)	0.930		
rs17658052	GG	371(93.9)	507(91.2)	1.00(ref)		1.00(ref)	
	AG	24(6.1)	47(8.5)	1.433(0.861–2.386)	0.166	1.419(0.852–2.365)	0.179
	AA	0(0.0)	2(0.4)	#	#	#	#
	AG + AA vs GG			1.494(0.900–2.479)	0.120	1.479(0.890–2.455)	0.131
	AA vs GG + AG			#	#	#	#
	G allele	766(97.0)	1061(95.4)	1.00(ref)			
	A allele	24(3.0)	51(4.6)	1.534(0.936–2.514)	0.087		

*: adjusted by age. #: could not be calculated

**Table 2:** Association between H19 SNPs and risks of lung adenocarcinoma and squamous cell lung cancer

H19		Controls (%)	Lung Adenocarcinoma					Squamous cell lung cancer				
			Cases (%)	OR (95% CI)	P value	OR (95% CI)*	*P* value	Cases (%)	OR (95% CI)	*P* value	OR (95% CI)*	*P* value
rs217727	CC	165(41.8)	141(38.0)	1.00(ref)		1.00(ref)		35(36.5)	1.00(ref)		1.00(ref)	
	CT	172(43.5)	169(45.6)	1.150(0.844–1.566)	0.376	1.152(0.846–1.570)	0.369	45(46.9)	1.233(0.755–2.015)	0.402	1.236(0.756–2.022)	0.399
	TT	58(14.7)	61(16.4)	1.231(0.805–1.881)	0.337	1.231(0.806–1.881)	0.337	16(16.7)	1.300(0.670–2.523)	0.437	1.315(0.676–2.556)	0.420
	CT + TT vs CC			1.170(0.876–1.563)	0.288	1.172(0.877–1.566)	0.283		1.250(0.788–1.983)	0.342	1.256(0.791–1.994)	0.335
	TT vs CT + CC			1.143(0.773–1.691)	0.502	1.142(0.773–1.689)	0.505		1.162(0.635–2.128)	0.626	1.173(0.640–2.153)	0.605
	C allele	502(63.5)	451(60.8)	1.00(ref)				115(59.9)	1.00(ref)			
	T allele	288(36.5)	291(39.2)	1.125(0.915–1.383)	0.265			77(3.6)	1.167(0.845–1.612)	0.384		
rs2107425	CC	140(35.4)	111(29.9)	1.00(ref)		1.00(ref)		27(28.1)	1.00(ref)		1.00(ref)	
	CT	185(46.8)	171(46.1)	1.166(0.843–1.613)	0.354	1.166(0.843–1.613)	0.354	43(44.8)	1.205(0.710–2.045)	0.489	1.183(0.696–2.011)	0.536
	TT	70(17.7)	89(24.0)	1.604(1.075–2.393)	0.021	1.602(1.074–2.390)	0.021	26(27.1)	1.926(1.046–3.545)	0.035	1.935(1.049–3.570)	0.035
	CT + TT vs CC			1.286(0.950–1.741)	0.104	1.286(0.949–1.741)	0.104		1.403(0.859–2.291)	0.176	1.387(0.848–2.268)	0.192
	CC vs CT + TT			1.465(1.031–2.082)	0.033	1.464(1.030–2.080)	0.034		1.724(1.026–2.898)	0.040	1.752(1.040–2.952)	0.035
	C allele	465(58.9)	393(53.0)	1.00(ref)				97(50.5)	1.00(ref)			
	T allele	325(41.1)	349(47.0)	1.271(1.038–1.555)	0.020			95(49.5)	1.401(1.021–1.923)	0.036		
rs2735469	CC	359(90.9)	340(91.6)	1.00(ref)		1.00(ref)		89(92.7)	1.00(ref)		1.00(ref)	
	CT	36(9.1)	29(7.8)	0.851(0.510–1.418)	0.535	0.851(0.510–1.418)	0.535	6(6.3)	0.672(0.275–1.645)	0.384	0.662(0.270–1.624)	0.368
	TT	0(0)	2(0.5)	#	#	#	#	1(1.0)	#	#	#	#
	CT + TT vs CC			0.909(0.550–1.503)	0.711	0.910(0.550–1.504)	0.712		0.784(0.338–1.821)	0.572	0.775(0.333–1.803)	0.554
	TT vs CC + CT			#	#	#	#		#	#	#	#
	C allele	754(95.4)	709(95.6)	1.00(ref)				184(95.8)	1.00(ref)			
	T allele	36(4.6)	33(4.4)	0.975(0.601–1.581)	0.918			8(4.2)	0.911(0.416–1.992)	0.815		
rs17658052	GG	371(93.9)	343(92.4)	1.00(ref)		1.00(ref)		82(85.4)	1.00(ref)		1.00(ref)	
	AG	24(6.1)	27(7.3)	1.217(0.689–2.150)	0.499	1.212(0.685–2.142)	0.509	13(13.5)	2.451(1.198–5.015)	0.014	2.411(1.175–4.946)	0.016
	AA	0(0.0)	1(0.3)	#	#	#	#	1(1.0)	#	#	#	#
	AG + AA vs GG			1.262(0.717–2.219)	0.419	1.256(0.714–2.211)	0.429		2.639(1.309–5.321)	0.007	2.587(1.279–5.229)	0.008
	AA vs GG + AG			#	#	#	#		#	#	#	#
	G allele	766(96.9)	713(96.1)	1.00(ref)			0.410	177(92.2)	1.00(ref)			
	A allele	24(3.0)	29(3.9)	1.298(0.749–2.251)	0.352			15(7.8)	2.705(1.390–5.262)	0.002		

*: adjusted by age

#: could not be calculated

We further explore the combined effects of H19 SNPs and cooking oil fume exposure on lung cancer risk (Table 3 and Table 4). For rs217727 polymorphism, the individuals with both CT/TT genotypes (risk genotype) and exposure to cooking oil fumes (risk factor) were more likely to develop lung cancer than those carrying risk genotype but without exposure to cooking fume or with exposure to cooking fume but carrying wild genotype, and the corresponding ORs (95%CIs) were 2.368 (1.409–3.978), 1.373 (0.898–2.098) and 1.921 (1.055–3.501), respectively. The similar results were also found in rs2107425 and rs2735469 polymorphisms, that was the combination of the risk genotypes of these SNPs and risk factor (cooking oil fume exposure) contributed to a higher risk of lung cancer (Table 3). Since adenocarcinoma is one of the most frequent subtype of lung cancer, with a higher incidence in women, we analyzed the combined effects of the SNPs in H19 gene and cooking oil fume exposure on lung adenocarcinoma susceptibility among female never smokers in Shenyang, China (Table 4). The combination of the risk genotype and exposure to cooking fume lead to a higher risk of lung adenocarcinoma than the individual effect of either risk genotype or exposure to cooking fume, suggesting that there may be interaction between these SNPs and cooking fume exposure, so we further explored the interaction on both additive and multiplicative scales through quantitative and statistically significant analysis (shown in Table 5).

**Table 3:** Combined effects of H19 SNPs and cooking oil fume exposure on lung cancer risk

SNP		Oil	Controls (%)	Cases (%)	OR (95%CI)^*^	*P* value
rs217727	CC	Non-exposure	86(32.3)	59(22.0)	1(ref)	
	CT + TT	Non-exposure	114(42.9)	109(40.7)	1.373(0.898–2.098)	0.143
	CC	Exposure	27(10.2)	36(13.4)	1.921(1.055–3.501)	0.033
	CT + TT	Exposure	39(14.6)	64(23.9)	2.368(1.409–3.978)	0.001
rs2107425	CC	Non-exposure	70(26.3)	43(16.0)	1(ref)	
	CT + TT	Non-exposure	130(48.9)	125(46.6)	1.550(0.985–2.439)	0.058
	CC	Exposure	25(9.4)	29(10.8)	1.852(0.960–3.575)	0.066
	CT + TT	Exposure	41(15.4)	71(26.5)	2.811(1.636–4.830)	< 0.001
rs2735469	CT + TT	Non-exposure	18(6.8)	10(3.7)	1(ref)	
	CC	Non-exposure	182(68.4)	158(59.0)	1.548(0.693–3.455)	0.286
	CT + TT	Exposure	9(3.4)	7(2.6)	1.388(0.395–4.876)	0.609
	CC	Exposure	57(21.4)	93(34.7)	2.906(1.253–6.744)	0.013
rs17658052	GG	Non-exposure	188(70.7)	150(56.0)	1(ref)	
	GA + AA	Non-exposure	12(4.5)	18 (6.7)	1.900(0.887–4.073)	0.099
	GG	Exposure	62(23.3)	94(35.1)	1.897(1.289–2.792)	0.001
	GA + AA	Exposure	4(1.5)	6(2.2)	1.920(0.531–6.948)	0.320

*: adjusted by age

**Table 4:** Combined effects of H19 SNPs and cooking oil fume exposure on lung adenocarcinoma risk

SNP		Oil	Controls (%)	Cases (%)	OR (95%CI)*	*P* value
rs217727	CC	Non-exposure	86(32.3)	49(24.9)	1(ref)	
	CT + TT	Non-exposure	114(42.9)	77 (39.1)	1.180(0.748–1.859)	0.477
	CC	Exposure	27(10.2)	23(11.7)	1.480(0.766–2.859)	0.243
	CT + TT	Exposure	39(14.6)	48(24.4)	2.147(1.239–3.721)	0.006
rs2107425	CC	Non-exposure	70(26.3)	36(18.3)	1(ref)	
	CT + TT	Non-exposure	130(48.9)	90(45.7)	1.342(0.827–2.177)	0.233
	CC	Exposure	25(9.4)	21(10.7)	1.614(0.796–3.273)	0.184
	CT + TT	Exposure	41(15.4)	50(25.4)	2.362(1.327–4.204)	0.003
rs2735469	CT + TT	Non-exposure	18(6.8)	7(3.6)	1(ref)	
	CC	Non-exposure	182(68.4)	119(60.4)	1.679(0.680–4.145)	0.261
	CT + TT	Exposure	9(3.4)	6(3.0)	1.697(0.438–6.570)	0.444
	CC	Exposure	57(21.4)	65(33.0)	2.917(1.136–7.492)	0.026
rs17658052	GG	Non-exposure	188(70.7)	114(57.9)	1(ref)	
	GA + GG	Non-exposure	12(4.5)	12 (6.1)	1.647(0.716–3.791)	0.241
	GG	Exposure	62(23.3)	68(34.5)	1.798(1.186–2.725)	0.006
	GA + AA	Exposure	4(1.5)	3(1.5)	1.268(0.278–5.781)	0.759

*: adjusted by age

**Table 5:** Measures of additive interaction between H19 SNPs and cooking oil fume exposure

SNP	Measure	Lung cancer		Lung adenocarcinoma	
		Estimate	95% CI	Estimate	95% CI
rs217727	RERI	0.074	−1.354-1.502	0.488	−0.797-1.773
	AP	0.031	−0.566-0.628	0.227	−0.326-0.780
	S	1.057	0.357–3.129	1.740	0.305–9.926
rs2107425	RERI	0.409	−1.127-1.944	0.405	−1.016-1.826
	AP	0.145	−0.374-0.665	0.172	−0.401-0.744
	S	1.291	0.465–3.589	1.424	0.357–5.683
rs2735469	RERI	0.970	−0.738-2.679	0.541	−1.573-2.654
	AP	0.334	−0.262-0.930	0.185	−0.588-0.929
	S	2.037	0.270–15.387	1.393	0.273–7.113
rs17658052	RERI	−0.877	−3.773-2.020	−1.177	−3.626-1.272
	AP	−0.456	−2.468-1.555	−0.928	−4.045-2.189
	S	0.512	0.032–8.226	0.1864	0.000–244.516

Table 5 showed the additive measures of biological interaction between SNPs in H19 gene and cooking fume exposure on lung cancer in never-smoking women of China. There are three measures of additive interaction as well as their confidence intervals. All measurements showed no significant interactions between the four SNPs and exposure to cooking fumes on the additive scale. Otherwise, based on logistic regression results, the gene-environment interactions on the multiplicative scale were not statistically significant (*P* values were 0.665, 0.928, 0.897 and 0.473 for rs217727, rs2107425, rs2735469 and rs17658052 in lung cancer, and 0.888, 0.903, 0.773 and 0.312 in lung adenocarcinoma).

To further investigate the relationship between H19 and cancer development, we summarized the effects of H19 on malignant phenotypes (including proliferation, differentiation, invasion, metastasis, and invasion) and its molecular mechanisms (see Additional file 1: Table S1). In addition, H19 has a variety of molecular functions, including transcriptional dyregulation, pre-mRNA alternative splicing, ceRNA role, epigenetic alterations and transition of cell phenotype through different signaling pathways covering EMT, Wnt and other pathways. Taken together, H19 exerted its molecular function in order to regulate the expression of related genes, which may affect carcinogenic signaling pathways, thereby promoting the progression of malignant carcinoma.

## Discussion

To the best of our knowledge, this study initiated the first survey to test associations between H19 polymorphisms (rs2107425, rs217727, rs2735469 and rs17658052) and lung cancer susceptibility, as well as the interactions between these polymorphisms and cooking oil fume exposure among female never smokers in Shenyang, China. The homozygous variant genotype of rs2107425 was thought to be associated with an increased risk of lung cancer. The individual effects of both SNP and cooking smoke exposure were much smaller than their combined effect on lung cancer. However, further analysis showed that the interactions on both additive and multiplicative models were not statistically significant.

Up to the present, some studies have shown the relationship between H19 polymorphisms and several types of cancer. Xia et al. indicated that there was a strong relationship between H19 polymorphisms (rs3741219 and rs217727) and the susceptibility of breast cancer in stratified analyses [17]. Another study found that the risk of bladder cancer in rs217727 AA genotype carriers was statistically significant increased compared with GG/GA genotype carriers [18]. Verhaegh et al. have found that H19 genetic polymorphisms (rs2839698 and rs2107425) might be associated with the susceptibility of bladder cancer in European Caucasians [19]. In the present study, we selected four common SNPs (rs217727, rs2107425, rs2735469, and rs17658052) in H19 gene to estimate the association between these variants and lung cancer susceptibility. We observe that rs2107425 polymorphism may be able to influence the risk of lung cancer in Chinese female never smokers. All of these suggested that H19 genetic variants might have an important effect on cancer susceptibility. However, to validate this conclusion, the researches in different ethnicities and the functional experiments are urgently needed.

H19, located in chromosome 11p15.5, is a paternally imprinted oncofetal lncRNA gene with oncogenic properties [20]. Previous studies have indicated the complex biological process of oncogenesis involves the participation of H19 [21, 22]. Jiang et al. showed that the invasion, angiogenesis, stemness, and tumorigenicity of glioblastoma cells were promoted by the increased expression of H19 lncRNA [23]. Despite the accumulation of evidence, the role of H19 in the molecular mechanism of tumorigenesis is still unclear. Evidence of genetic variants in lncRNAs that modified the risk of tumors continued to emerge [24, 25]. In addition, multiple SNPs have been identified associated with cancer susceptibility by genome-wide association studies (GWASs). However, there still have some limits in the clarification of causal SNPs and the deep mining of GWAS data. In recent years, these have attracted the attention of researchers [26–28]. In the present study, the association between H19 polymorphisms and lung cancer susceptibility was analyzed based on our previous GWAS results. Ultimately, we need sophisticated experimental design and implementation in order to not only further reveal the mystery of lncRNA but also effectively address diagnostic/prognostic biomarkers and therapeutic targets for cancer.

Indeed, lncRNAs may have crucial roles in some important aspects such as drug response and toxicity. Previous studies have shown that these two SNPs were involved in platinum-based chemotherapy responses in lung cancer. Gong et al. indicated that H19 rs2839698 had a relationship with platinum-based chemotherapy response, and H19 rs2107425 was associated with platinum-based chemotherapy response in patients with small cell lung cancer [29]. We found that H19 rs2107425 and rs2839698 were associated with severe gastrointestinal toxicity, and rs2839698 in lung cancer patients with severe hematologic toxicity have accepted GP regimen [30]. The report also showed that there was a significant association between overexpression of H19 and the poor survival of lung cancer patients [31].

A known risk factor of lung cancer is cigarette smoking. However, the public and researchers need to be aware of the impact of other environmental risk factors on lung cancer. The epidemiologic characteristics of lung cancer in Chinese women have been discovered by the researchers, that is, they smoke very little but have lung cancer is relatively frequent, so the epidemiologic characteristics of lung cancer cannot be fully explained by cigarette smoking. In this sense, the ideal subjects for detecting unknown factors in lung cancer are the females who never smoke. In order to examine risk factors and genetic susceptibility of lung cancer in Chinese never-smoking females, our research team has been devoting themselves. In previous studies, the associations between cooking oil fume exposure and lung cancer risk women who never smoke have been noted. In the present study, so as to obtain some clues about the biological role that the gene-environment interaction played in the development of lung cancer, the interaction of both lncRNA H19 SNPs and cooking oil fume with the susceptibility of lung cancer was evaluated. However, we found that the interactions of four SNPs and cooking oil fume exposure were not statistically significant through the statistical analysis on both additive scale and multiplicative scale.

There are several limitations of the present research. First, since all subjects were recruited from the hospitals, we cannot avoid selection bias. Second, when collecting demographic data, some risk factors associated with the case group may be taken by the investigators, such as the status of cooking oil fume, which as far as known are associated with the case group while in a manner that is incomparable to the control group. Finally, since all subjects of our study are limited to Chinese never-smoking females, the sample size is not very large. In future study, so as to verify the conclusion in different races, we need a larger population of lung cancer. For the present result is just a statistical estimation, the biological validity of lncRNA and their SNPs are required in further functional studies.

In summary, this study showed that the single nucleotide polymorphisms in H19 gene might play vital roles in lung cancer development. There is a positive significance in our study to explore the pathogenesis of lung cancer, and to some degree, provide clues for diagnostic biomarkers and therapeutic targets of lung cancer in future.

## Conclusion

The polymorphism rs2107425 in H19 gene is associated with the risk of lung cancer among female never smokers in Shenyang, China.

## Additional file

Additional file 1: **Table S1.** Research advances of H19 roles in human cancers (**indicates** increase; **indicates** decrease). (DOCX 239 kb)

## Abbreviations

AP: The attributable proportion due to interaction
CI: Confidence interval
GWAS: Genome-wide association study
lncRNA: Long non-coding RNA
OR: Odds ratio
RERI: The relative excess risk due to interaction
S: The synergy index
SNP: Single nucleotide polymorphism
