# Supplementary material for: Polymorphisms in the H19 gene and the risk of lung Cancer among female never smokers in Shenyang, China
Source: BMC Cancer. 2018 Sep 15;18:893. doi: 10.1186/s12885-018-4795-6 (PMC6139161; doi:10.1186/s12885-018-4795-6)
Supplement: Supplementary file 1 — Table S1. Research advances of H19 roles in human cancers (indicates increase; indicates decrease). (DOCX 239 kb) [file 12885_2018_4795_MOESM1_ESM.docx]

Additional file 1

Table S1 Research advances of H19 roles in human cancers ( **indicates** increase; **indicates** decrease)

| **Cancer type** | **Expression effect** | **Clinical features of up-regulated H19** | **Transition of cell phenotype of H19 inhibition** | **Carcinogenic mechanisms** | **References** |
| --- | --- | --- | --- | --- | --- |
| Gastric cancer | Up-regulated | OS; TNM stage | proliferation / invasion | H19 siRNA and miR-141 could decrease miR-675 expression and induce Igf1r/Igf2 expression; MiR-141 inhibitor restores H19 siRNA function; miR-141 could bind to a sequence in H19 and suppress H19 expression; H19-Derived miR-675 interacts with RUNX1; H19 acts as a ceRNA to regulate human epidermal growth factor receptor expression by sequestering let‑7c. | [[1-4](#_ENREF_1)] |
| Bladder cancer | Up-regulated | LNM / DM / tumor size | proliferation / metastasis / invasion / migration | H19-derived miR-675 regulates p53 activation; H19 regulates ID2 expression; H19 interacts with EZH2 and E-cadherin via Wnt/β-catenin pathway; H19 regulates EMT and cell metastasis by miR-29b-3p as ceRNA and relieve the suppression for DNMT3B. | [[5-9](#_ENREF_5)] |
| Prostate cancer | Down-regulated | - | migration | H19/miR-675 axis could inhibit cell metastasis by targeting TGFBI. | [[10](#_ENREF_10)] |
| Gallbladder cancer | Up-regulated | OS; TNM stage / LNM | invasion / proliferation; cell cycle arrest in G0/G1 phase | H19 regulates FOXM1 expression by binding miR-342-3p; Knockdown of H19 could upregulate miR-194-5p levels and decrease miR-194-5p targeting AKT2 expression; H19/miR-194-5p/AKT2 axis regulatory network. | [[11-13](#_ENREF_11)] |
| Lung cancer | Up-regulated | TNM stage / tumor size; OS | the number of cells in G2/M stage; proliferation / migration / invasion; apoptosis | C-Myc up-regulated H19 expression and strengthen the activity of H19 promoter; H19 interacts with miR-107; H19-elevated LIN28B interacts with miR-196b; H19 activates STAT3 signaling via sponging miR-17; H19 promotes EMT by targeting miR-484. | [[14-17](#_ENREF_14)] |
| Colorectal cancer | Up-regulated | differentiation / TNM stage; OS / DFS | proliferation / migration / invasion | H19 overexpression promotes EMT; H19 serves as a molecular ‘sponge’ for miR-138 and miR-200a, targeting mesenchymal marker genes including vimentin, ZEB1, and ZEB2; H19 regulates β-Catenin activity via influencing CDK8 expression; H19 regulates RB1-E2F1 activity; H19 obstructs the recruitment of eIF4A3 to their mRNA; H19 acts as ceRNA by binding to miR-138 to upregulate HMGA1 expression; H19 could competitively bind to miR-200a and derepresse β-Catenin expression; H19/miR-29b-3p/PGRN axis promotes EMT by Wnt pathway. | [[18-23](#_ENREF_18)] |
| Thyroid cancer | Up-regulated | LNM / TNM stage / tumor size; OS | proliferation / migration / invasion | H19 competitively binds miR-17-5p to regulate YES1 expression. | [[24](#_ENREF_24), [25](#_ENREF_25)] |
| Ovarian cancer | Up-regulated | - | migration / invasion; apoptosis | The H19/let-7 axis is antagonized by metformin-induced DNA methylation; H19 promotes TGF-β-induced EMT by sponging miR-370-3p. | [[26-28](#_ENREF_26)] |
| Hepatocellular carcinoma | Down-regulated | HBV infection / AFP; DFS | invasion / metastasis | H19 could influence the EMT; H19 upregulates the miR-200 family by interacting with the HnRNP U/PCAF/RNA PolII complex and promoting histone acetylation. | [[29](#_ENREF_29)] |
| Nasopharyngeal carcinoma | Up-regulated | - | invasion / metastasis | H19 regulates EZH2 expression through interacting with miR-630. | [[30](#_ENREF_30)] |
| Esophageal squamous cell carcinoma | Up-regulated | LNM / tumor invasion depth | proliferation / metastasis; cell cycle arrest in G0/G1 phase; | H19 DMR methylation could contribute to tumour progression via IGF2 imprinting; Knockdown of H19 enhances E-cadherin expression, whereas decreases expression of vimentin and metastasis-associated protein such as MMP-9. | [[31](#_ENREF_31), [32](#_ENREF_32)] |
| Osteosarcoma | Up-regulated | - | migration / invasion | H19 promotes metastasis through upregulation of ZEB1 and ZEB2 and competitively binding the miR-200 family. H19 involves in the nuclear factor-κB pathway. | [[33](#_ENREF_33), [34](#_ENREF_34)] |
| Breast cancer | Up-regulated | ER / PR / LNM | proliferation / invasion / migration | H19 overexpression relieved the inhibition of miR-152 on DNMT1 expression; H19 binds to miR-152 as a ceRNA; H19/miR-675 could induce up-regulation of tyrosine kinase receptors and activation of the downstream AKT and ERK pathways. | [[35-37](#_ENREF_35)] |
| Melanoma | Up-regulated | TNM stage / LNM / DM | proliferation / migration / invasion; | H19 promotes the glucose metabolism and growth of melanoma cell by sponging miR-106a-5p and up-regulating E2F3 expression. | [[38-40](#_ENREF_38)] |
| Pancreatic ductal adenocarcinoma | Up-regulated | differentiation | proliferation / viability ; apoptosis; cell cycle arrest in G0/G1 phase; | E2F-1 could influence oncogenic effects of H19; H19 could derive miR-675 interacts with E2F-1. | [[41](#_ENREF_41), [42](#_ENREF_42)] |
| Clear cell renal cell carcinoma | Up-regulated | differentiation / DM / LNM / tumor size;  OS | migration / invasion | H19 could regulate expression of endogenous miR-29a-3p targeted E2F1 by competitively binding miR-29a-3p; | [[43](#_ENREF_43), [44](#_ENREF_44)] |
| Glioma | Up-regulated | tumor size/stage; OS | proliferation / migration/ invasion / metastasis | H19 interacts with miR-140 by targeting inhibitor of apoptosis-stimulating protein of p53 (iASPP); H19 inhibits microRNA-29a. | [[45-47](#_ENREF_45)] |

OS: overall survival; DFS: disease-free survival; LNM: lymph node metastasis; DM: distant metastasis; HBV: hepatitis B virus; AFP: 𝛼-fetoprotein; ER: estrogen receptor; PR: progesterone receptor.

**References：**

1. Zhou X, Ye F, Yin C, Zhuang Y, Yue G, Zhang G: The Interaction Between MiR-141 and lncRNA-H19 in Regulating Cell Proliferation and Migration in Gastric Cancer. Cellular physiology and biochemistry : international journal of experimental cellular physiology, biochemistry, and pharmacology 2015, 36(4):1440-1452.

2. Liu G, Xiang T, Wu QF, Wang WX: Long Noncoding RNA H19-Derived miR-675 Enhances Proliferation and Invasion via RUNX1 in Gastric Cancer Cells. Oncology research 2016, 23(3):99-107.

3. Wei Y, Liu Z, Fang J: H19 functions as a competing endogenous RNA to regulate human epidermal growth factor receptor expression by sequestering let7c in gastric cancer. Molecular medicine reports 2018, 17(2):2600-2606.

4. Zhang EB, Han L, Yin DD, Kong R, De W, Chen J: c-Myc-induced, long, noncoding H19 affects cell proliferation and predicts a poor prognosis in patients with gastric cancer. Medical oncology 2014, 31(5):914.

5. Liu C, Chen Z, Fang J, Xu A, Zhang W, Wang Z: H19-derived miR-675 contributes to bladder cancer cell proliferation by regulating p53 activation. 2016, 37(1):263-270.

6. Luo M, Li Z, Wang W, Zeng Y, Liu Z, Qiu J: Upregulated H19 contributes to bladder cancer cell proliferation by regulating ID2 expression. The FEBS journal 2013, 280(7):1709-1716.

7. Luo M, Li Z, Wang W, Zeng Y, Liu Z, Qiu J: Long non-coding RNA H19 increases bladder cancer metastasis by associating with EZH2 and inhibiting E-cadherin expression. Cancer letters 2013, 333(2):213-221.

8. Lv M, Zhong Z, Huang M, Tian Q, Jiang R, Chen J: lncRNA H19 regulates epithelial-mesenchymal transition and metastasis of bladder cancer by miR-29b-3p as competing endogenous RNA. Biochimica et biophysica acta 2017, 1864(10):1887-1899.

9. Zhu Z, Xu L, Wan Y, Zhou J, Fu D, Chao H, Bao K, Zeng T: Inhibition of E-cadherin expression by lnc-RNA H19 to facilitate bladder cancer metastasis. Cancer biomarkers : section A of Disease markers 2018, 22(2):275-281.

10. Zhu M, Chen Q, Liu X, Sun Q, Zhao X, Deng R, Wang Y, Huang J, Xu M, Yan J et al: lncRNA H19/miR-675 axis represses prostate cancer metastasis by targeting TGFBI. The FEBS journal 2014, 281(16):3766-3775.

11. Wang SH, Ma F, Tang ZH, Wu XC, Cai Q, Zhang MD, Weng MZ, Zhou D, Wang JD, Quan ZW: Long non-coding RNA H19 regulates FOXM1 expression by competitively binding endogenous miR-342-3p in gallbladder cancer. Journal of experimental & clinical cancer research : CR 2016, 35(1):160.

12. Wang SH, Wu XC, Zhang MD, Weng MZ, Zhou D, Quan ZW: Long noncoding RNA H19 contributes to gallbladder cancer cell proliferation by modulated miR-194-5p targeting AKT2. Tumour biology : the journal of the International Society for Oncodevelopmental Biology and Medicine 2016, 37(7):9721-9730.

13. Wang SH, Wu XC, Zhang MD, Weng MZ, Zhou D, Quan ZW: Upregulation of H19 indicates a poor prognosis in gallbladder carcinoma and promotes epithelial-mesenchymal transition. American journal of cancer research 2016, 6(1):15-26.

14. Cui J, Mo J, Luo M, Yu Q, Zhou S, Li T, Zhang Y, Luo W: c-Myc-activated long non-coding RNA H19 downregulates miR-107 and promotes cell cycle progression of non-small cell lung cancer. International journal of clinical and experimental pathology 2015, 8(10):12400-12409.

15. Zhang E, Li W, Yin D, De W, Zhu L, Sun S, Han L: c-Myc-regulated long non-coding RNA H19 indicates a poor prognosis and affects cell proliferation in non-small-cell lung cancer. Tumour biology : the journal of the International Society for Oncodevelopmental Biology and Medicine 2016, 37(3):4007-4015.

16. Ren J, Fu J, Ma T, Yan B, Gao R, An Z, Wang D: LncRNA H19-elevated LIN28B promotes lung cancer progression through sequestering miR-196b. Cell cycle (Georgetown, Tex) 2018.

17. Huang Z, Lei W, Hu HB: H19 promotes non-small-cell lung cancer (NSCLC) development through STAT3 signaling via sponging miR-17. 2018, 233(10):6768-6776.

18. Han D, Gao X, Wang M, Qiao Y, Xu Y, Yang J, Dong N, He J, Sun Q, Lv G et al: Long noncoding RNA H19 indicates a poor prognosis of colorectal cancer and promotes tumor growth by recruiting and binding to eIF4A3. Oncotarget 2016, 7(16):22159-22173.

19. Yang Q, Wang X, Tang C, Chen X, He J: H19 promotes the migration and invasion of colon cancer by sponging miR-138 to upregulate the expression of HMGA1. International journal of oncology 2017, 50(5):1801-1809.

20. Yang W, Ning N: The lncRNA H19 Promotes Cell Proliferation by Competitively Binding to miR-200a and Derepressing beta-Catenin Expression in Colorectal Cancer. 2017, 2017:2767484.

21. Liang WC, Fu WM, Wong CW, Wang Y, Wang WM, Hu GX, Zhang L, Xiao LJ, Wan DC, Zhang JF et al: The lncRNA H19 promotes epithelial to mesenchymal transition by functioning as miRNA sponges in colorectal cancer. Oncotarget 2015, 6(26):22513-22525.

22. Ohtsuka M, Ling H, Ivan C, Pichler M, Matsushita D, Goblirsch M, Stiegelbauer V, Shigeyasu K, Zhang X, Chen M et al: H19 Noncoding RNA, an Independent Prognostic Factor, Regulates Essential Rb-E2F and CDK8-beta-Catenin Signaling in Colorectal Cancer. EBioMedicine 2016, 13:113-124.

23. Ding D, Li C, Zhao T, Li D, Yang L, Zhang B: LncRNA H19/miR-29b-3p/PGRN Axis Promoted Epithelial-Mesenchymal Transition of Colorectal Cancer Cells by Acting on Wnt Signaling. Molecules and cells 2018, 41(5):423-435.

24. Liu L, Yang J, Zhu X, Li D, Lv Z, Zhang X: Long noncoding RNA H19 competitively binds miR-17-5p to regulate YES1 expression in thyroid cancer. The FEBS journal 2016, 283(12):2326-2339.

25. Yang Z, Lu Y, Xu Q, Tang B, Park CK, Chen X: HULC and H19 Played Different Roles in Overall and Disease-Free Survival from Hepatocellular Carcinoma after Curative Hepatectomy: A Preliminary Analysis from Gene Expression Omnibus. Dis Markers 2015, 2015:191029.

26. Yan L, Zhou J, Gao Y, Ghazal S, Lu L, Bellone S, Yang Y, Liu N, Zhao X, Santin AD et al: Regulation of tumor cell migration and invasion by the H19/let-7 axis is antagonized by metformin-induced DNA methylation. Oncogene 2015, 34(23):3076-3084.

27. Zhu Z, Song L, He J, Sun Y, Liu X, Zou X: Ectopic expressed long non-coding RNA H19 contributes to malignant cell behavior of ovarian cancer. International journal of clinical and experimental pathology 2015, 8(9):10082-10091.

28. Li J, Huang Y, Deng X, Luo M, Wang X, Hu H, Liu C, Zhong M: Long noncoding RNA H19 promotes transforming growth factor-beta-induced epithelial-mesenchymal transition by acting as a competing endogenous RNA of miR-370-3p in ovarian cancer cells. OncoTargets and therapy 2018, 11:427-440.

29. Zhang L, Yang F, Yuan JH, Yuan SX, Zhou WP, Huo XS, Xu D, Bi HS, Wang F, Sun SH: Epigenetic activation of the MiR-200 family contributes to H19-mediated metastasis suppression in hepatocellular carcinoma. Carcinogenesis 2013, 34(3):577-586.

30. Li X, Lin Y, Yang X, Wu X, He X: Long noncoding RNA H19 regulates EZH2 expression by interacting with miR-630 and promotes cell invasion in nasopharyngeal carcinoma. Biochemical and biophysical research communications 2016, 473(4):913-919.

31. Gao T, He B, Pan Y, Gu L, Chen L, Nie Z, Xu Y, Li R, Wang S: H19 DMR methylation correlates to the progression of esophageal squamous cell carcinoma through IGF2 imprinting pathway. Clinical & translational oncology : official publication of the Federation of Spanish Oncology Societies and of the National Cancer Institute of Mexico 2014, 16(4):410-417.

32. Tan D, Wu Y, Hu L, He P, Xiong G, Bai Y, Yang K: Long noncoding RNA H19 is up-regulated in esophageal squamous cell carcinoma and promotes cell proliferation and metastasis. Diseases of the esophagus : official journal of the International Society for Diseases of the Esophagus 2017, 30(1):1-9.

33. Li M, Chen H, Zhao Y, Gao S, Cheng C: H19 Functions as a ceRNA in Promoting Metastasis Through Decreasing miR-200s Activity in Osteosarcoma. DNA and cell biology 2016, 35(5):235-240.

34. Zhao J, Ma ST: Downregulation of lncRNA H19 inhibits migration and invasion of human osteosarcoma through the NF-kappaB pathway. Molecular medicine reports 2018, 17(5):7388-7394.

35. Li Z, Li Y, Li Y, Ren K, Li X, Han X, Wang J: Long non-coding RNA H19 promotes the proliferation and invasion of breast cancer through upregulating DNMT1 expression by sponging miR-152. Journal of biochemical and molecular toxicology 2017, 31(9).

36. Vennin C, Spruyt N, Dahmani F, Julien S, Bertucci F, Finetti P, Chassat T, Bourette RP, Le Bourhis X, Adriaenssens E: H19 non coding RNA-derived miR-675 enhances tumorigenesis and metastasis of breast cancer cells by downregulating c-Cbl and Cbl-b. Oncotarget 2015, 6(30):29209-29223.

37. Zhang K, Luo Z, Zhang Y, Zhang L, Wu L, Liu L, Yang J, Song X, Liu J: Circulating lncRNA H19 in plasma as a novel biomarker for breast cancer. Cancer biomarkers : section A of Disease markers 2016, 17(2):187-194.

38. Luan W, Zhou Z, Ni X, Xia Y, Wang J, Yan Y, Xu B: Long non-coding RNA H19 promotes glucose metabolism and cell growth in malignant melanoma via miR-106a-5p/E2F3 axis. 2018, 144(3):531-542.

39. Liao Z, Zhao J, Yang Y: Downregulation of lncRNA H19 inhibits the migration and invasion of melanoma cells by inactivating the NFkappaB and PI3K/Akt signaling pathways. Molecular medicine reports 2018, 17(5):7313-7318.

40. Shi G, Li H, Gao F, Tan Q: lncRNA H19 predicts poor prognosis in patients with melanoma and regulates cell growth, invasion, migration and epithelial-mesenchymal transition in melanoma cells. OncoTargets and therapy 2018, 11:3583-3595.

41. Ma L, Tian X, Wang F, Zhang Z, Du C, Xie X, Kornmann M, Yang Y: The long noncoding RNA H19 promotes cell proliferation via E2F-1 in pancreatic ductal adenocarcinoma. Cancer Biol Ther 2016, 17(10):1051-1061.

42. Ma L, Tian X, Guo H, Zhang Z, Du C, Wang F, Xie X, Gao H, Zhuang Y, Kornmann M et al: Long noncoding RNA H19 derived miR-675 regulates cell proliferation by down-regulating E2F-1 in human pancreatic ductal adenocarcinoma. Journal of Cancer 2018, 9(2):389-399.

43. He H, Wang N, Yi X, Tang C, Wang D: Long non-coding RNA H19 regulates E2F1 expression by competitively sponging endogenous miR-29a-3p in clear cell renal cell carcinoma. Cell & bioscience 2017, 7:65.

44. Wang L, Cai Y, Zhao X, Jia X, Zhang J, Liu J, Zhen H, Wang T, Tang X, Liu Y et al: Down-regulated long non-coding RNA H19 inhibits carcinogenesis of renal cell carcinoma. Neoplasma 2015, 62(3):412-418.

45. Zhao H, Peng R, Liu Q, Liu D, Du P, Yuan J, Peng G, Liao Y: The lncRNA H19 interacts with miR-140 to modulate glioma growth by targeting iASPP. Archives of biochemistry and biophysics 2016, 610:1-7.

46. Jia P, Cai H, Liu X, Chen J, Ma J, Wang P, Liu Y, Zheng J, Xue Y: Long non-coding RNA H19 regulates glioma angiogenesis and the biological behavior of glioma-associated endothelial cells by inhibiting microRNA-29a. Cancer letters 2016, 381(2):359-369.

47. Zhang T, Wang YR, Zeng F, Cao HY, Zhou HD, Wang YJ: LncRNA H19 is overexpressed in glioma tissue, is negatively associated with patient survival, and promotes tumor growth through its derivative miR-675. European review for medical and pharmacological sciences 2016, 20(23):4891-4897.
